# Fixation techniques in lower extremity correction osteotomies and fractures in mild-to-severe osteogenesis imperfecta patients: evaluation of the results and complications

**DOI:** 10.1186/s13018-023-03917-z

**Published:** 2023-06-16

**Authors:** Abdulsamet Emet, Engin Turkay Yilmaz, Murat Danisman, Cemalettin Aksoy, Guney Yilmaz

**Affiliations:** 1 Department of Orthopedics and Traumatology, Yuksek Ihtisas University, Private Liv Hospital, Turan Gunes Bulv. Koz Apt No: 41/22 Cankaya, Ankara, Turkey; 2 Department of Orthopedics and Traumatology, Hacettepe University Faculty of Medicine, Ankara, Turkey; 3 Department of Orthopedics and Traumatology, Giresun University Prof. Dr. A. Ilhan Ozdemir Hospital, Giresun, Turkey

**Keywords:** Osteogenesis imperfecta, Plate, Intramedullary fixation, Osteotomy, Fracture

## Abstract

**Introduction:**

Osteogenesis imperfecta is a genetic disorder leading to multiple fractures and deformities. Intramedullary rods have been used in the surgical treatment of osteogenesis imperfecta for decades. Complication rates reported by current techniques have been high. This study aimed to examine the results of intramedullary fixation combined with plate and screw technique in patients with osteogenesis imperfecta compared to isolated intramedullary fixation.

**Methods:**

Between 2006 and 2020, forty patients who had surgical treatment for deformities or fractures of the femur, tibia or both with at least two years of follow-up after surgery were included in the study. Patients were divided into groups according to fixation methods. Group 1 was intramedullary fixation only (Titanium Elastic Nail [TEN], Rush Pin, and Fassier-Duval Rod), and Group 2 was intramedullary fixation combined with plate and screws. Medical records and follow-up radiographs were reviewed to evaluate healing and callus formation, types of complications and infection rates.

**Results:**

The total number of operated lower extremities of these forty patients was 61 (45 femur and 16 tibia). The mean age of the patients was 9.3 ± 4.6 years. Mean follow-up duration of the patients was 4.4 ± 1.7 years. Thirty-seven (61%) were in Group 1, and 24 (39%) were in Group 2. There was no statistically significant difference in callus formation time between Group 1 and Group 2 (*p* = 0.67). Complications occurred in 21 of 61 surgeries. While 17 of these complications were in Group 1, 4 were in Group 2 (*p* = 0.01).

**Conclusion:**

Intramedullary fixation combined with the plate and screw technique in children with osteogenesis imperfecta is successful considering the complications and revision requirements.

## Introduction

Osteogenesis imperfecta (OI) is a genetic disorder caused by alterations in collagen type 1, characterized by low bone mass and poor strength [1]. The severity of the disease ranges from mildly affected individuals with minimal deformities to lethally affected patients with multiple fractures and functional disabilities. Fractures begin to appear in the first years after walking age and lead to progressive deformities [2]. The important feature of fractures in OI is that they develop at the apex of the deformity, resulting in more distorted extremities [3].

The multidisciplinary management strategy is mainstay for this multi-systemic disease, including physiotherapy, rehabilitation, and orthopedic surgery [4]. Additionally, anti-osteoporosis medications are used to strengthen the bones [5–7]. The key step in the orthopedic treatment of patients with OI is to prevent fracture and deformity by providing structural support. Therefore, surgical decision and the selection of implants for the patients have become the focus of orthopedic surgeons.

The gold-standard fixation technique is telescopic intramedullary nailing. In cases of narrowed canals or patients with young age, the choice of intramedullary fixation is non-telescopic nails, which are titanium elastic nails and rush pins [8]. However, the use of these implants has not yielded satisfactory results [9, 10]. In 1963, Bailey designed the Dubow–Bailey rod with a T-piece on the male part and a curved end on the female part [11]. Based on the Dubow–Bailey rod, the Sheffield telescopic rod has been designed, which effectively reduces the risk of proximal migration seen in Dubow–Bailey rod [12]. However, insertion of the rod via arthrotomy has been shown to damage the joint [13]. It was a turning point when François Fassier and Pierre Duval designed a new telescopic rod with two screw tips with the advantages of avoiding knee or ankle arthrotomy and reducing soft tissue injury [14], though the risk of rod migration is still known to be high. Additionally, the major limitation of any intramedullary fixation is rotational and longitudinal instability [15, 16]. Therefore, a fully satisfactory type of fixation could not be obtained.

The objective of the current study was to examine the results of intramedullary fixation combined with plate and screw technique in patients with osteogenesis imperfecta compared to isolated intramedullary fixation. This study assessed the following conditions: callus formation and healing, deformity recurrence, implant migration, infection, and revisions.

## Material and methods

Approval of the local ethics committee was obtained before data collection. Written informed consent was obtained from the legal guardian of each patient. A total of 90 patients followed in the Pediatric Endocrinology Department that diagnosis was based mainly on clinical and radiological features, between 2006 and 2020, were evaluated retrospectively. Among these 90 patients, a total of 40 patients were included in the study. Inclusion criteria were determined as being under the age of eighteen when the first surgical treatment was performed in Orthopedics and Traumatology Department, having surgical treatment for deformities or fractures of the femur, tibia or both, having at least two years of follow-up post-surgery in Orthopedics and Traumatology Department, being followed up simultaneously by the Pediatric Endocrinology department and receiving appropriate bisphosphonate and vitamin D therapy. A total of 50 patients whose records could not be reached, who was followed up by only cast immobilization, who did not undergo lower extremity surgery due to deformity correction or fracture, and who discontinued follow-ups or were followed up for less than two years were excluded from the study. While 16 of 40 patients included in the study were Sillence Type III, remaining 24 patients were Sillence Type IV.

**Fig. 1:**
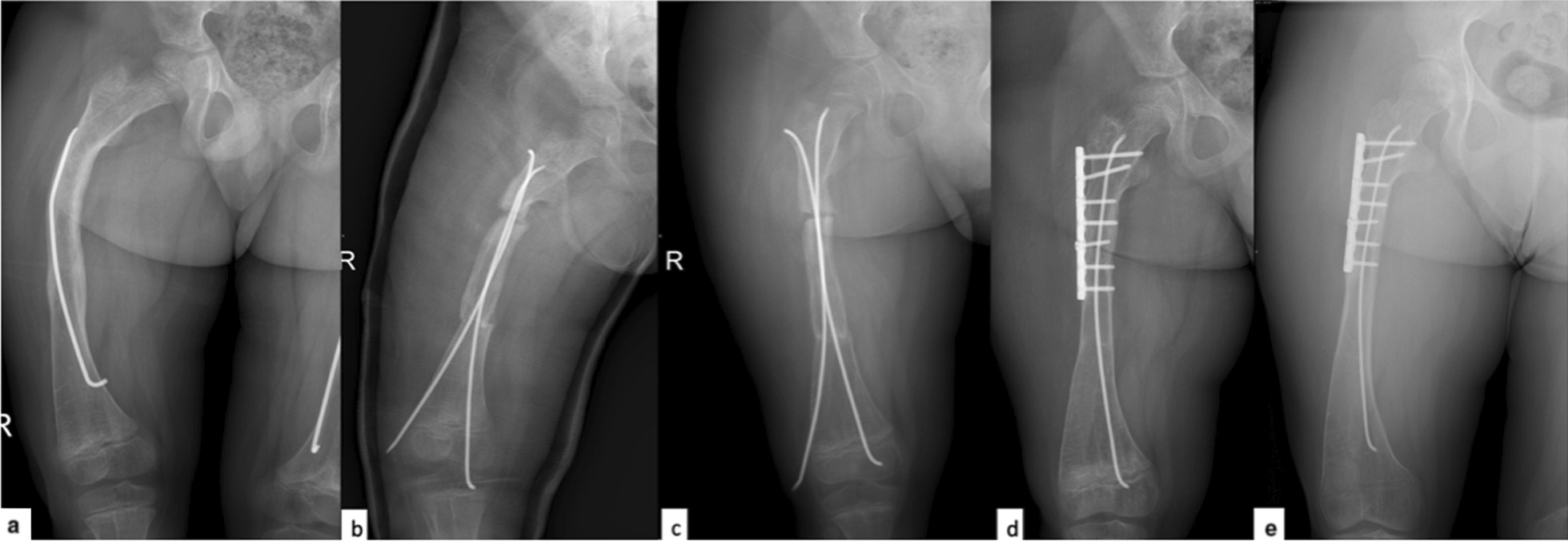
Patient with intramedullary fixation only **a** preoperative radiograph, **b** postoperative first-day radiograph, **c** postoperative sixth-month radiograph, union in the distal osteotomy of the patient but nonunion was seen in the proximal osteotomy, **d** second-year follow-up radiograph after revision surgery and plate fixation. Total union was seen and deformity recurrence was not observed, **e** third-year follow-up radiograph after revision surgery and plate fixation. Deformity recurrence was not observed

**Fig. 2:**
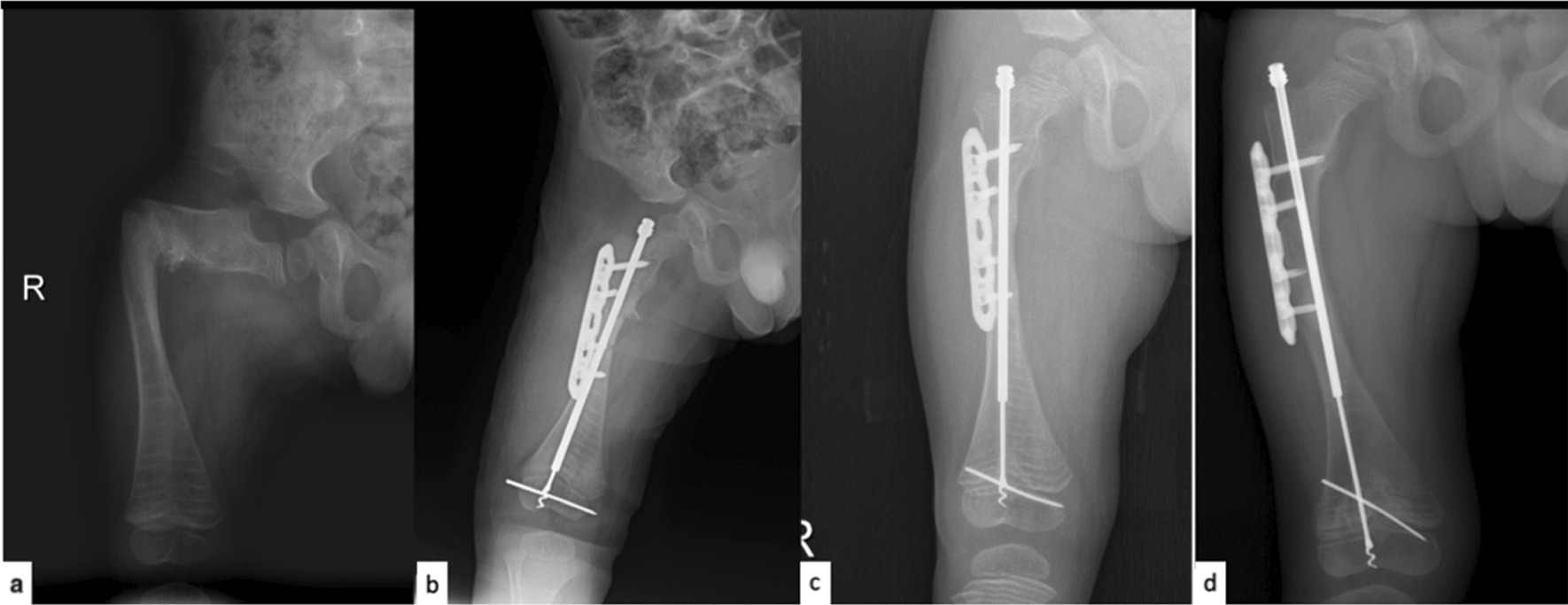
Patient with intramedullary fixation combined with plate and screws **a** preoperative radiograph, **b** postoperative first-day radiograph, **c** second-year follow-up radiograph, **d** third-year follow-up radiograph

Age, sex, surgery site, fixation method, callus formation, complication and complication types, and infection rates were noted. Patients were divided into groups according to fixation methods. Group 1 was intramedullary fixation only (Titanium Elastic Nail [TEN], Rush Pin, and Fassier-Duval Rod [D-Scope Telescopic Nail, TST Medical Devices, Istanbul, Turkey]) (Fig. 1), and Group 2 was intramedullary fixation combined with plate and screws (Fig. 2). The plate application site was in the extraperiosteal, submuscular space. Plate and screws were kept if there were no issues related to plate. In this study, none of the supplemental plate and screws were removed from the patients during the entire follow-up. The patients were followed up with a routine control protocol. Partial weight bearing was allowed when callus was seen in the radiographs of the patients in the follow-up, and after the union was assured, they were allowed to walk with total weight bearing.

Statistical analysis was performed in the Statistical Package for the Social Sciences (SPSS) 22 package program. Descriptive statistical results are presented as medians (minimum-maximum) and frequencies with percentages. The analyses were the Mann‒Whitney U test, Chi-square test, and Fisher’s exact test. The results had a 95% confidence interval, and the statistical significance was at the *p* < 0.05 level.

## Results

Forty participants (24 males and 16 females) with a mean age of 9.3 ± 4.6 years (boys 9.7 ± 1.2, girls 10.7 ± 1.7) were included in the study. The mean age of the patients in Group 1 was 10 ± 4.9 years, and that in Group 2 was 8 ± 3.5 years. The mean follow-up of patients in Group 1 was 4 ± 2 years, and that in Group 2 was 3.5 ± 1.2 years. The total number of operated lower extremities of these 40 patients was 61 (45 femur and 16 tibia). Thirty-seven (61%) were in Group 1, and 24 (39%) were in Group 2. No statistically significant difference was present in the evaluation of the age, sex, and follow-up of the patients, (*p* > 0.05). The clinical characteristics of the two groups are shown in Table 1.

**Table 1:** Comparison of clinical characteristics between Group 1 and Group 2

	All participants	Group 1	Group 2	*p*
Age (y)	9.3 ± 4.6	10 (4–17)	8 (3–17)	0.07
Sex (M/F)	24/16	14/10	10/6	0.6
Follow-up (y)	4.4 ± 1.7	4.0 (2–9)	3.5 (2–6)	0.19
Operated limb (tibia/femur)	16/45	8/29	8/16	0.2
Callus formation (week)	5.75 (± 1.92)	5.83 (± 2.16)	5.62 (± 1.49)	0.67

Complications were present in 21 of the 61 surgeries performed. While 17/21 were in Group 1, 4/21 were in Group 2 (*p* = 0.01). Of the 21 complications, 5 were seen after tibia surgery and 16 after femoral surgery. There were no statistically significant differences in the incidence of complications of tibia surgery in Group 1 (3/8, 38%) and Group 2 (2/8, 25%) (*p* = 0.5). However, among the femur operations, complications developed in 14 of 29 surgeries in Group 1, while there were only two complications in 16 surgeries in Group 2. (*p* = 0.01). The most common complication in Group 1 was implant migration (27%). Additionally, 16% of patients in Group 1 had deformity recurrence. In Group 2, implant migration was 8%, and deformity recurrence was 8%. The number of lower extremities operated for fracture were fifteen (13 femur and 2 tibia). Twelve lower extremities were present in Group 1 (11 femur and 1 tibia), and three lower extremities were present in Group 2 (2 femurs and 1 tibia) due to fracture fixation. Complications were observed in three patients who underwent surgery for fracture in Group 1 (25%, 3 migration), while no complications were observed in Group 2. A statistically significant difference was found between Group 1 and Group 2 patients who underwent surgery for correction osteotomy (*p* = 0.011). There was no statistically significant difference between Group 1 and Group 2 patients who underwent surgery for fracture (*p* = 0.484). There were no statistically significant differences in the incidence of total complications between limbs operated for fracture and osteotomy in Group 1 (*p* = 0.077). There were no statistically significant differences in the incidence of total complications between limbs operated for fracture and osteotomy in Group 2 (*p* = 0.563). The types and numbers of complications seen in Group 1 and Group 2 are shown in Table 2.

**Table 2:** Results and complications in Group 1 and Group 2

	Group 1 (n = 37)		Group 2 (n = 24)	
	Fracture	Osteotomy	Fracture	Osteotomy
Number (lower limbs)	12	25	3	21
Implant migration	3	7	0	2
Deformity recurrence	0	6	0	2
Nonunion	0	1	0	0
Total complication	3	14	0	4
Callus formation (week)	5.91(± 3.47)	5.80 (± 1.22)	5 (± 1)	5.71(± 1.55)
Revision surgery	2	8	0	4

Revision surgery required 4/4 (100%) (2 implant migration and 2 deformity recurrence) and 10/17 (59%) (7 implant migration, 2 deformity recurrence and 1 nonunion) complications in Group 2 and Group 1, respectively (*p* = 0.16). The median time to revision was nine months (0–40) and eight months (3–13) in Group 1 and Group 2, respectively (*p* = 0.7).

Lower extremities were evaluated as callus is present or absent. Since the postoperative follow-up X-ray timing of the patients varied, the average value was obtained according to the time the callus was first seen on the radiograph. The mean callus formation time of the patients in Group 1 was 5.83 (± 2.16) weeks and that in Group 2 was 5.62 (± 1.49) weeks. There was no statistically significant difference in callus formation time between Group 1 and Group 2 (*p* = 0.67). Callus formation was observed in all patients in both groups, except for one patient in Group 1. This patient in group 1 was the patient who underwent osteotomy after deformity recurrence and was fixed with non-telescoping rods. Since nonunion was observed in the follow-up, the patient underwent to revision surgery at sixth month and fixation with plate and screws was performed in addition to intramedullary fixation. Total union was seen and deformity recurrence was not observed at third-year follow-up (Fig. 1).

We did not observe any complications related to the plate, and no loosening or pull-out of screws was observed. In the 12th-month follow-up, 2-mm pull-out was observed in only one proximal side screw of a plate that applied to proximal femur in one patient, while no additional retraction in this screw or pull-out in the other screws of this plate was observed in the follow-ups. No additional intervention was performed on this patient due to no mechanical symptoms and patient did not have complaints due to the screw and continued to be followed up.

Superficial wound infection developed in a patient in Group 2 who underwent intramedullary nail and plate fixation due to a femur fracture. The patient was treated with oral antibiotics and did not need additional debridement or implant revision surgery.

## Discussion

In the current study, we present that intramedullary fixation combined with the plate and screw technique in children with osteogenesis imperfecta is successful in terms of complications and revisions. The implant migration rate and deformity recurrence reduced. In Group 1, the total complication rate was 45% (17/37), and in Group 2, it was 16% (4/24). Overall, this rate decreased by 2.8 times.

Intramedullary rods (telescoping/non-telescoping) protect the long bones from deformities and refractures in the early postoperative period. The long bones of patients with OI are thin and have a small canal diameter, and this physical feature therefore requires the insertion of thin intramedullary rods that can fit into the bone in surgical treatment, but this is not ideal to achieve effective stabilization [15, 16]. Also, as a common complication, failure of telescoping and migration of the intramedullary rod have been reported in several clinical series [13, 17]. In our study, the most common complication observed in the intramedullary fixation group was implant migration (27%). If failure of telescoping and proximal migration of the rod occur, the implant acts as a non-elongating rod. However, due to the continuing growth of the patient and affected bone, there is a predisposition to deformity and fracture with loss of this support proximal or distal to the rod [17, 18]. As the proximal rod migration is generally associated with osteotomy/fracture gap and angular deformity, obtaining additional stability becomes even more important. Spahn et al. reported higher revision rates in the first 48 months using non-telescoping rods [19]. In our study, the number of patients who underwent revision due to deformity recurrence in group 2 was only two lower limbs, and this rate decreased according to the literature. The results indicate that additional fixation of plate and screws combined with intramedullary rod prevents migration and recurrence of deformity.

This is the first study as far as we have investigated in English literature that examines the results of the comparison of intramedullary fixation combined with plate and screw technique versus isolated intramedullary fixation in patients with osteogenesis imperfecta. Furthermore, this is the study with the highest number of patients treated with intramedullary fixation combined with plate and screws. Considering similar studies, in 2015, Cho et al. suggested the technique of plate fixation, in which screws hold a single cortex together combined with an intramedullary fixation procedure [20]. In this study, unicortical locking plate fixation offers an additional fixation method in OI patients. This additional stability offered increases the chances of complete healing of the fracture or osteotomy. Furthermore, in 2018, Franzone et al. published the short-term results of plate application in addition to the intramedullary nail in 11 OI patients and suggested that plate fixation achieved better rotational stability and union at the osteotomy or fracture site with no refracture [21].

Another issue to keep in mind is infection rates. The number of infections may increase due to higher implant load and incisions. However, the infection rates in the literature are between 0 and 5% in patients with isolated intramedullary fixation [9, 22, 23]. In our patient group, infection was present in only one patient and was considered a superficial wound infection treated with oral antibiotics without any additional intervention. The total rate was consistent with the literature. Increasing metallic hardware or the number of the patients’ incisions did not increase infection rates.

Our study has limitations, such as being a retrospective study with variability in the reason for surgery and the operation on different bones. Since OI is a rare disorder, our study had a small number of patients. Studies with more patients with homogenous groups are needed. Another limitation of the study is the fact that the follow-up period of the patients was not long enough to elucidate long-term complications of additional plate fixation.

In conclusion, intramedullary fixation combined with the plate and screw technique in children with osteogenesis imperfecta is a valuable treatment option. Further follow-up will provide more insight into long-term outcomes.

## Data Availability

The data used and/or analyzed during the current study are available from the corresponding author upon reasonable request.
